# Time to recovery and its predictors following traumatic injuries among injured victims in Dessie Comprehensive Specialized Hospital, North East of Ethiopia, 2022: a retrospective follow-up study

**DOI:** 10.1186/s12873-024-00960-9

**Published:** 2024-03-18

**Authors:** Lehulu Tilahun, Mulusew Zeleke, Birhanu Desu, Kirubel Dagnew, Aytenew Nega, Endalk Birrie, Nathan Estifanos, Akele Tegegne, Asresu Feleke

**Affiliations:** 1 College of Medicine and Health Sciences, Department of Emergency and Ophthalmic Nursing, Wollo University, PO Box 1145, Dessie, Ethiopia; 2 College of Medicine and Health Sciences, Department of Adult Health Nursing, Wollo University, Dessie, Ethiopia; 3 College of Medicine and Health Sciences, Department of Comprehensive Nursing, Wollo University, Dessie, Ethiopia; 4 College of Medicine and Health Sciences, Department of Pediatrics and Child Health, Wollo University, Dessie, Ethiopia; 5 College of Medicine and Health Sciences, Department of Emergency and Critical Care Nursing, Dilla University, Dilla, Ethiopia

**Keywords:** Trauma, Injury, Predictor, Associated factor, Recovery, Time

## Abstract

**Background:**

Injuries are an extremely important public health problem worldwide. Despite being largely preventable and despite government efforts, injuries continue to be a major public health issue. Thus, the study tends to evaluate the time to recovery and its predictors for traumatic injuries.

**Methods:**

A hospital-based retrospective follow-up study was used. A total of 329 medical charts were actually reviewed. Traumatic injury victims from January 1, 2018–December 31, 2022 were included, and a simple random sampling technique was utilized. The data was gathered by reviewing medical charts. Data was coded and entered into Epi-Data Manager version 4.6.0.4 statistical software and further analyzed using STATA version 17. Descriptive statistics were performed to see the frequency distribution of variables. A Kaplan-Meier survival estimate and log rank test were performed to plot the overall survival curve and compare the difference in recovery among predictor categories, respectively. A model fitness test was done by using the Cox-Snell residual test and Harrell’s C concordance statistic. Finally, a Cox proportional hazard model was fitted to determine the effect of predictors on recovery time from traumatic injuries.

**Results:**

The median time to recovery of traumatic injuries was 5 days (IQR: 3–10 days), with an overall incidence density of 8.77 per 100 person-days of observation. In the multivariable cox proportional regression model, variables such as being male (AHR: 0.384, 95%CI: 0.190–0.776, *P*-value: 0.008), the Glasgow coma scale of 13–15 (AHR: 2.563, 95%CI: 1.070–6.139, *P*-value: 0.035), intentional injury (AHR: 1.934, 95%CI: 1.03–3.632, *P*-value: 0.040), mild traumatic brain injury (AHR: 2.708, 95%CI: 1.095–6.698, *P*-value: 0.031), and moderate traumatic brain injury (AHR: 2.253, 95%CI: (1.033–4.911, *P*-value: 0.041) were statistically significant variables.

**Conclusions:**

The median recovery time for traumatically injured respondents was 5 days. Independent predictors such as the Glasgow coma scale, time taken for surgical management, intent of injury, and traumatic brain injury were statistically significant with time to recovery from trauma.

**Supplementary Information:**

The online version contains supplementary material available at 10.1186/s12873-024-00960-9.

## Introduction

A traumatic event or accident is defined as any event or accident that causes physical or psychological harm. Traumas may occur intentionally or unintentionally through traffic accidents, falling accidents, burns, or assaults. With unacceptable dalliance, this requires emergent and urgent trauma care. According to a World Health Organization (WHO) report, more than 14,000 people are killed every day due to injuries. In other words, traumatic accidents claim the lives of 5 million or more people worldwide, mainly because of road traffic accidents (RTAs), which account for 9% of all deaths. This figure is 1.7 times higher than the combined deaths resulting from HIV/AIDS, malaria, and tuberculosis. From here, less developed states account for an estimated 85% of deaths and 90% of annual disability-adjusted life years (DALYs) [1–3]. For many years, traumatic injuries have not received the expected attention from the world’s health concerns, even though they can be preventable [4].

The median recovery time for traumatic injuries was reported differently in different parts of the world. The median time to recovery pinpointed was four (4) days in the United States [5], five (5) days in Iran [6], six (6) days in Canada [7], and twenty-four (24) days in Kenya [8].

Traumatic injuries are six of the top fifteen causes of death in children aged 15 to 14. It is assumed that the main causes of injuries identified are RTAs, assault, drowning, and falls. Despite efforts to reduce the impact of RTA, there are few efforts to combat other causes of trauma that have a negative impact on the lives of the community [9, 10].

In spite of the big challenge of traumatic injuries, the burden of injury is not fully understood, especially in low- and middle-income countries (LMICs). There have been few studies, and the majority of them are clinical-based studies, which do not fully represent the true picture of the population because most injured victims cannot get to the clinical area for a variety of reasons, including the cost of treatment, a lack of transportation or an ambulance, and distance from health services. This leads to most trauma search literature revealing developed countries with established trauma systems [11]. Injuries still become a major public health issue despite being largely preventable and despite government efforts, including in Ethiopia [12].

In Ethiopia, more than 50% of surgical emergencies are the result of trauma. 2000 individuals are killed in motor vehicle accidents per year. There are 48% pedestrians, 45% passengers, and 7% drivers [13]. A study revealed in Ethiopia found that 93.8% of respondents survived, with a median time to recovery of four (4) days and a total length of stay (LOS) of 1–105 days [14].

As per the knowledge of the investigator, there is very little information about the time to recovery from trauma among injury patients in Ethiopian hospitals. In addition, research done in an Ethiopian hospital was mainly focused on surgical ward admitted patients. Even though the majority of the studies were mainly intended to examine the outcome and prevalence of traumatic injuries, the median time of survival and its related predictors were not well assessed. Thus, this research intends to assess the time to recovery and its predictors among injured patients at the emergency department of Dessie Comprehensive Specialized Hospital.

## Methods

### Study setting and period

The study was conducted at Dessie Comprehensive Specialized Hospital, found in Amhara Regional State. Dessie town is located 401 km from the Centr, Addis Ababa, and 495 km from Bahirdar, the capital city of the Amhara region. Dessie Comprehensive Specialized Hospital is one of six government referral hospitals in the Amhara region. The hospital is under the supervision of the Regional Health Bureau. The hospital will serve 5 million people. In the hospital, a total of 400 traumatically injured patients visit per month. That means, on average, 14 traumatically injured respondents came to the hospital per day. The study period was from January 1, 2018 to December 31, 2022.

### Study design

A hospital-based retrospective follow-up study design was used among injured victims in Dessie Comprehensive Specialized Hospital, Ethiopia, in 2022.

### Source and study populations

The source population included all traumatically injured patients treated at Dessie Comprehensive Specialized Hospital in 2022. All traumatically injured patients treated at Dessie Comprehensive Specialized Hospital during the study period were considered the study population.

### Inclusion and exclusion criteria

All traumatically injured adult victims treated at Dessie Comprehensive Specialized Hospital were eligible for the study. Incomplete charts with baseline medical data, mainly for variables not recorded like emergency department (ED) admission time, duration of waiting at the ED, and ED discharge date, were excluded from the study.

### Sample size determination and sampling procedure

The power and sample size determinations for the survival study were done using STATA version 17 with stpower log rank and the Freedman method. So, with a 95% confidence interval and 80% power, the overall sample size to be used is 352 subjects, with a 5% censoring rate and a 10% non-response rate (Additional file 1). For doing that, different literature predictors with their corresponding hazard ratio (HR), estimated number of events, and estimated initial sample sizes were calculated. A simple random sampling procedure was used to select subjects from the sampling frame after checking and excluding ineligible medical charts.

### Data collection procedure

A review of eligible trauma charts of traumatically injured victims at Dessie Comprehensive Specialized Hospital (at the ED, ICU (intensive care unit), and surgical ward, as well as at the temporary trauma center) was used for data collection. Five data collectors (including a BSC emergency nurse) and one supervisor (with a background in public health) were assigned as supervisors. With some modifications, a research questionnaire could be adapted from the WHO 2002 injury surveillance guideline [15] and other literature [14, 16]. The event for this study was the time to recover of traumatically injured patients in the emergency department.

### Operational definition

According to this study, *“Time to Recovery”:* Status of a Traumatic Victim from Time of Admission to the Hospital to Discharge Time with Recovery in Days, *“Event”:* Those with medical charts of trauma patients labelled “discharged with recovery” were among those who visited Dessie Comprehensive Specialized Hospital. *“Censored”:* Those of traumatically injured patients with death, not being discharged, referral, refusal to treatment, or an unknown outcome status. *“The Glasgow Coma Scale (GCS)”* is a clinical scale used to measure level of consciousness objectively after traumatic brain injury (TBI) by measuring eye opening, verbal response, and motor response. It is scaled as severe TBI (3–8), moderate TBI (9–12), and mild TBI (13–15) [17]. *“Emergency Department/ED”:* First entry and critical hospital area that services emergency victims (trauma/RTA, cardiac emergency, respiratory emergency, poisoning, etc.).

### Data quality control

To increase data quality, a 1-day training for data collectors and supervisors with regard to data collection and a questionnaire were provided to clear up any difficulties. *Investigators* and supervisors supervised the data collection on a daily basis. The filled tools were checked for completeness by the investigators and supervisor, and corrective measures will be provided. To assure the quality of the data, it was entered by the principal investigator.

### Data processing and analysis

Data were entered into Epi-Data Manager statistical software (version 4.6.0.4) and analysed using STATA version 17. Findings were displayed through tables and charts. Descriptive statistics were done through frequencies, means, standard deviations, medians, and other measures. Kaplan-Meier (KM) survival estimates were drawn to observe the total survival curve and categorical predictors.

A log rank test was run to compare the difference in recovery among different predictor categories.

After the proportional hazard assumption was tested with a graphical test (a log-log plot of survival) and Schoenfeld’s global test of survival, a further regression model was selected. In addition, multicollinearity was tested by the Pearson correlation coefficient and the variance inflation factor (VIF). To determine the significant effect of predictors on the event of interest, the *P* value, adjusted hazard ratio (AHR), and 95% confidence interval were calculated.

Model fitness was tested using the Cox-Snell residual and Harrell’s C concordance statistic.

## Results

### General description of the study population

From January 1, 2018 to December 31, 2022, a total of 24,000 traumatic patients visited Dessie Comprehensive Specialized Hospital for the study of traumatic injuries. Among them, 352 study subjects were randomly sampled, and a total of 329 study respondents were actually investigated in the study.

### Socio-demographic and baseline trauma characteristics

Men make up more than three-quarters of the study participants (283, or 86.02%). The mean age of study respondents was 32.89 ± 13.91 years (min: 13, max: 90). Over half of the participants, 179 (54.41%), were rural residents. Three hundred twenty (97.26%) of the research subjects said vital information was obtained.

The majority of the 133 (40.43%) study subjects were awake, followed by 75 (22.8%) who were unconscious and 72 (21.88%) who were pain sensitive. One hundred forty-two (44.38%) subjects were < = 8 GCS, 9–12 GCS, and 13–15 GCS levels, which contained 131 (39.82%) and 52 (15.81%), respectively. Most of the 280 (85.11%) study participants had no history of complications. Above three-fourths (314; 95.44%) of subjects had no history of complications. Among those with complications, almost half (46.67%) had cardiovascular system disease, followed by respiratory system disease and endocrine comorbidities, which accounted for 4 (26.67%) and 3 (20%), respectively. More than half of the 10 patients (66.67%) had two or more complications (Table 1).

**Table 1:** Socio-demographic and baseline trauma characteristics of study respondents at Dessie Comprehensive Specialized Hospital, North East Ethiopia, 2022

Variables	Category	Frequency	Percent (%)
Sex	Male	283	86.02
	Female	46	13.98
Age	All	32.89 ± 13.91 (Min:13, Max: 90)	
Residency	Urban	150	45.59
	Rural	179	54.41
Vital Sign taken at arrival	Yes	320	97.26
	No	9	2.74
Level of Consciousness	Alert	133	40.43
	Verbally Responsive	72	21.88
	Responsive for pain	49	14.89
	Unconscious	75	22.80
GCS of Participants	13–15	52	15.81
	9–12	131	39.82
	< = 8	146	44.38
Previous trauma history	Yes	49	14.89
	No	280	85.11
Comorbidities	Yes	15	4.56
	No	314	95.44
If “yes” types of comorbidities	Respiratory disease	4	26.67
	Cardiovascular disease	7	46.67
	Neurological disease	1	6.67
	Endocrine disease	3	20
Number of comorbidities	One	5	33.33
	Two or more	10	66.67

### Clinical characteristics of trauma respondents

As per the study, most (319, or 97.26%) of the study participants received management before admission. Two hundred twenty-eight (69.3%) of study subjects had no complications, and the rest, 101 (30.7%), had complications. Most of the 228 (69.30%) research respondents replied that less than 24 h of time were taken for surgical management after admission. The ED was the primary source of admission for more than half of the subjects, followed by trauma centers, which were mentioned by 80 (24.93%). Above half (205, 62.31%) of respondents had received an intentional type of injury, and 124, 37.69%, had gotten an unintentional type of trauma. The majority (199, or 97.07%) of intentionally injured victims were victims of interpersonal injury. Two hundred twenty-one (67.17%) participants used ambulances as their mode of transport, followed by taxis (explained by 99, or 30.09%).

The majority of the clients had a bullet injury as the mechanism of injury, followed by a stab injury, an RTA, and a falling accident, accounting for 95 (28.88%), 71 (21.58%), and 37 (11.25%), respectively. Over half of RTA victims (55, or 77.46%) were occupants. The majority of 106 (32.22%) subjects developed concussions, followed by organ system injuries in 87 (26.44%) participants. One hundred sixty-two (49.24%) of subjects replied that the head was the site of injury, followed by the chest, abdomen, and extremities, which were described by 68 (20.67%), 48 (14.59%), and 41 (12.46%) of study participants, respectively. Of head injury victims, the majority developed moderate TBI (traumatic brain injury) and severe TBI, which accounted for 92 (56.79%) and 58 (35.68%).

Two hundred twenty-nine (69.6%) of trauma respondents did not receive mechanical ventilation. Above three-fourths, 261 (79.33%) subjects received clinical, laboratory, and imaging investigations, followed by clinical and laboratory investigations, which accounted for 48 (14.59%).

**Fig. 1:**
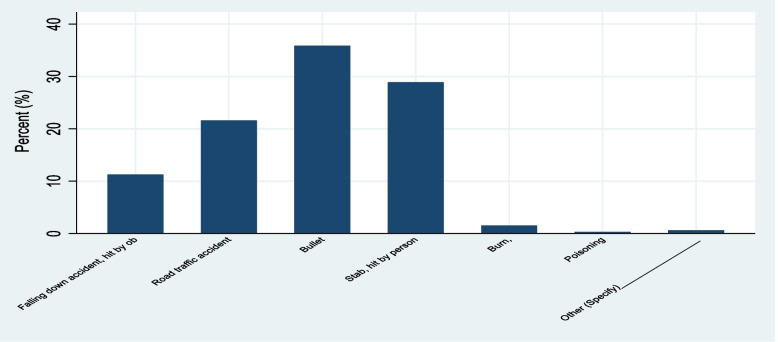
Mechanisms of trauma of study respondents at Dessie Comprehensive Specialized Hospital, North East Ethiopia, 2022

**Fig. 2:**
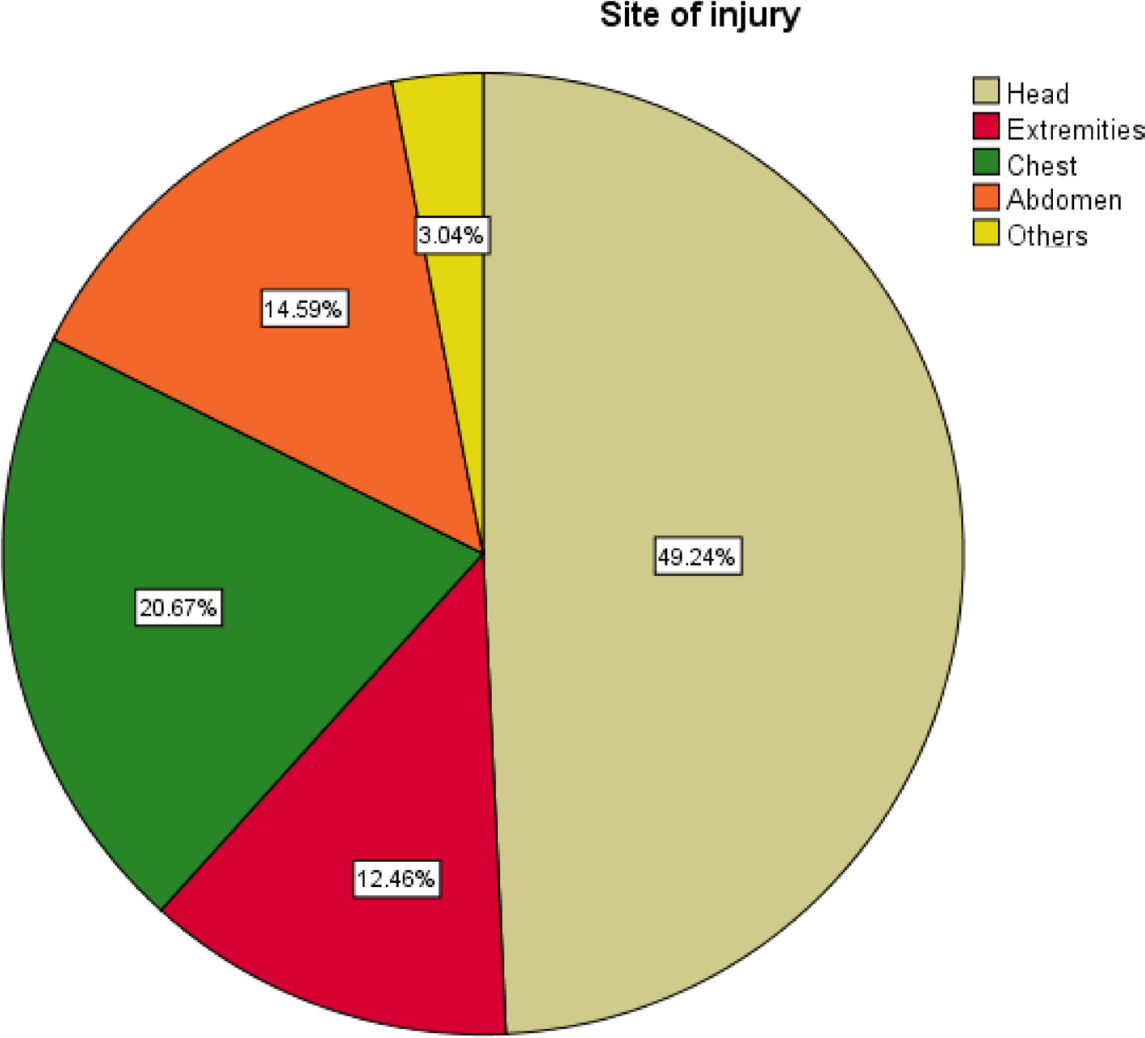
Site of trauma of study respondents at Dessie Comprehensive Specialized Hospital, North East Ethiopia, 2022.  Brown (49.24%): indicated head injured victims.  Red (12.46%): extremities injury.  Green (20.67%): chest injuries.  Semi-red (14.59%): abdominal injuries.  Yellow (3.04%): others- neck, bladder, maxilla

Three hundred sixteen (316) (96.05%) of participants received treatments before reaching the ED. Before arriving at the ED, the vast majority (311, or 98.42%) were treated by trained personnel (Table 2, Figs. 1 and 2).

**Table 2:** Clinical characteristics of trauma study respondents at Dessie Comprehensive Specialized Hospital, North East Ethiopia, 2022

Variables	Category	Frequency	Percent (%)
Managements given before admission	Yes	319	97.26
	No	9	2.74
Complications present	Yes	101	30.7
	No	228	69.3
Time taken for surgical management after admission	< 24 h	228	69.30
	25-48 h	82	24.92
	> 48 h	19	5.78
Source of admission	Emergency Department (ED)	183	55.62
	Intensive Care Unit	1	0.3
	Trauma center	80	24.93
	Surgical ward	42	12.77
	Operation Room	21	6.38
	Others (Specify)^a^	2	0.61
Intent of Injury	Intentional	205	62.31
	Unintentional	124	37.69
Mode of Transport	Ambulance	221	67.17
	Taxi	99	30.09
	On foot	9	2.74
Type of intentional Injury	Self-harm	5	2.44
	Interpersonal	199	97.07
	Others (Specify)^b^	1	0.49
Mechanisms of injury	Falling down accident, hit by object	37	11.25
	Road traffic accident	71	21.58
	Bullet	118	35.87
	Stab, hit by a person	95	28.88
	Burn	5	1.52
	Poisoning	1	0.3
	Others (Specify)^b^	2	0.61
If RTA, role of a participant	Pedestrian	15	21.13
	Occupant	55	77.46
	Motorcyclist	1	1.41
Nature of trauma	Cuts, lacerations	84	25.53
	Burn	6	1.82
	Concussion	106	32.22
	Sprain, Strain,	8	2.43
	Fracture	38	11.55
	Organ system involved	87	26.44
Number of organs injured	Single	254	77.2
	Multiple	75	22.8
Site of injury	Head	162	49.24
	Extremities	41	12.46
	Chest	68	20.67
	Abdomen	48	14.59
	Others (Specify)^c^	10	3.04
If head injury, its severity	Mild TBI	12	7.41
	Moderate TBI	92	56.79
	Sever TBI	58	35.80
Received mechanical ventilation	Yes	96	29.18
	No	229	69.6
	Others (Specify)^d^	4	1.22
Type of investigations done	Clinical & Laboratory	20	6.08
	Clinical & Imaging	48	14.59
	Clinical, Laboratory & imaging	261	79.33
Treatment given before ED	Yes	316	96.05
	No	13	3.95
If Yes, type of treatment provided	No care	3	0.95
	Treatment by non-trained person	2	2
	Treatment by trained person	311	98.42

^a^Orthopedic ward

^b^Ox injury

^c^Neck, bladder, maxilla

^d^Against to treatment

### Time to recovery for respondents from traumatic injuries

More than half of the 232 (70.52%) respondents with a 95%CI of 65.34%–75.21%) recovered from traumatic injuries, while the remaining 97 (29.48%) with a 95%CI of 24.79%–34.66%) were censored. Among the censored observations, 37 (39.78%) were deaths, 35 (37.63%) were referrals, and 17 (18.28%) were refused treatment. It was found that the median recovery of traumatically injured respondents was 5 days, with an interquartile range (IQR) of 3–10 days.

**Fig. 3:**
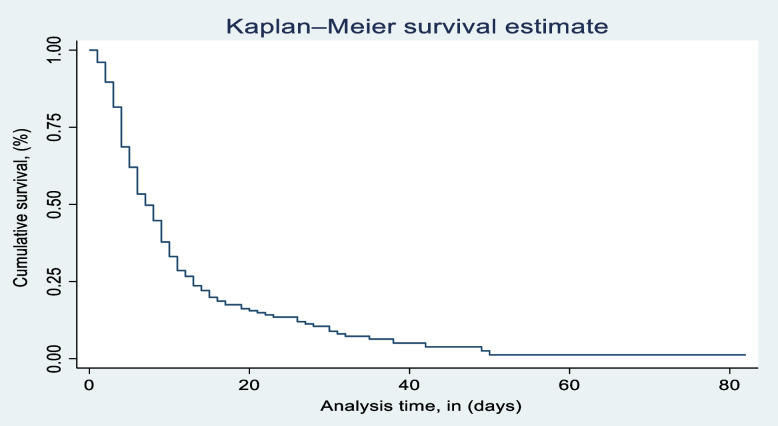
Kaplan-Meier survival estimate of study respondents at Dessie Comprehensive Specialized Hospital, North East Ethiopia, 2022

The total number of person-day observations was 2,646.3 days, with a total recovery rate of 8.77 per 100 person-days (95% CI: 7.70–9.97). According to the Kaplan-Meier estimates of 90-day recovery for trauma respondents, the graph plot decreases as the duration of time in days increases. In a 90-day life table analysis of trauma subjects, the highest day of recovery was observed on the 10th day, with a recovery of 33.13% (95% C.I.: 27.41%–38.95%), and on the 20th day, with a recovery of 15.59% (95% C.I.: 10.99%–20.92%). For instance, the recovery rate of traumatically injured respondents at day ten was 7.33 per 100 person days of observation (Tables 3 and 4, Fig. 3).

**Table 3:** Site of trauma of study respondents at Dessie Comprehensive Specialized Hospital, North East Ethiopia, 2022

Variables	Category	Frequency	Percent (%)
Length of stay in days	IQR = 5–10	Median: 5	
Out come	Event	232	70.52
	Censored	97	29.48
If Censored, what’s next	Died	37	39.78
	Referral	35	37.63
	Refused to treatment	17	17
	Others^a^	4	4.3

^a^Discharge, not-improved, left due to war, & absconded

**Table 4:** Kaplan-Meier survivor function by time indicator of study respondents at Dessie Comprehensive Specialized Hospital, North East Ethiopia, 2022

Time	Beg. Total	Fail	Survivor function
0.3	329	0	1
10	88	182	0.3313 (0.2741–0.3895)
20	25	34	0.1559 (0.1099–0.2092)
30	13	9	0.0890 (0.0515–0.1390)
40	5	4	0.0510 (0.0214–0.0999)
50	2	3	0.0127 (0.0013–0.0580)
60	2	0	0.0127 (0.0013–0.0580)
70	2	0	0.0127 (0.0013–0.0580)
80	2	0	0.0127(0.0013–0.0580)

### Survival difference test

**Fig. 4:**
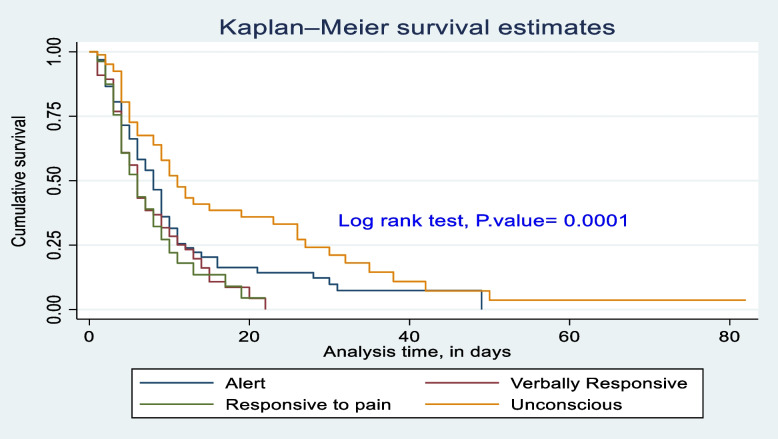
Kaplan-Meier survival estimate of study respondents by level of consciousness at Dessie Comprehensive Specialized Hospital, North East Ethiopia, 2022

**Fig. 5:**
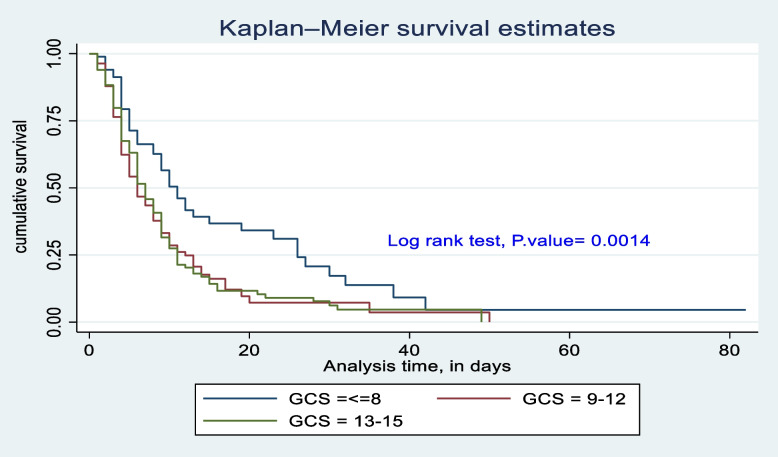
Kaplan-Meier survival estimate of study respondents by Glasgow coma scale at Dessie Comprehensive Specialized Hospital, North East Ethiopia, 2022

**Fig. 6:**
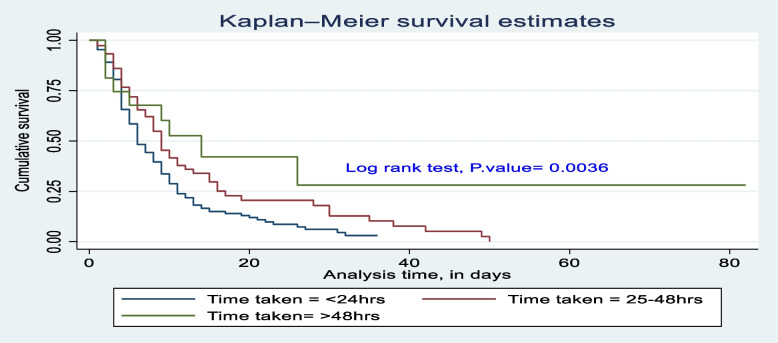
Kaplan-Meier survival estimate of study respondents by time taken for surgical management at Dessie Comprehensive Specialized Hospital, North East Ethiopia, 2022

**Fig. 7:**
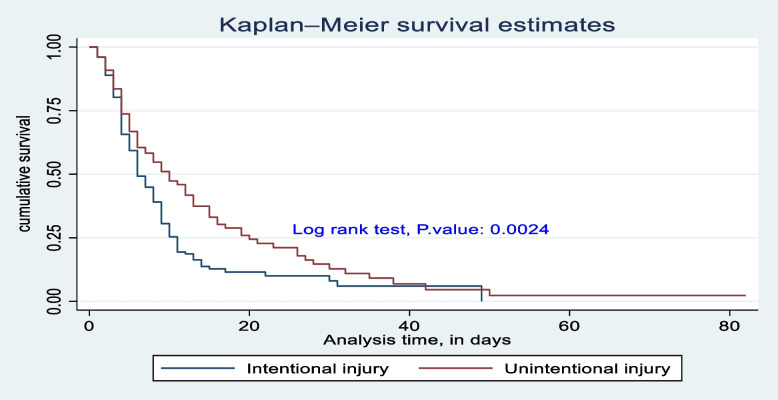
Kaplan-Meier survival estimate of study respondents by intent of injury at Dessie Comprehensive Specialized Hospital, North East Ethiopia, 2022

**Fig. 8:**
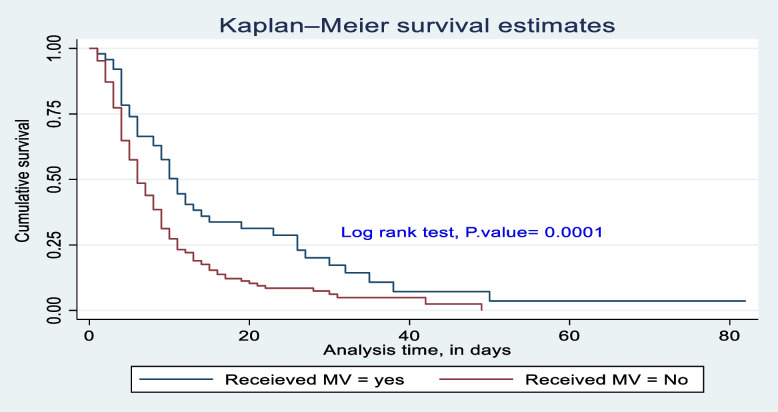
Kaplan-Meier survival estimate of study respondents by received mechanical ventilation or not at Dessie Comprehensive Specialized Hospital, North East Ethiopia, 2022

**Fig. 9:**
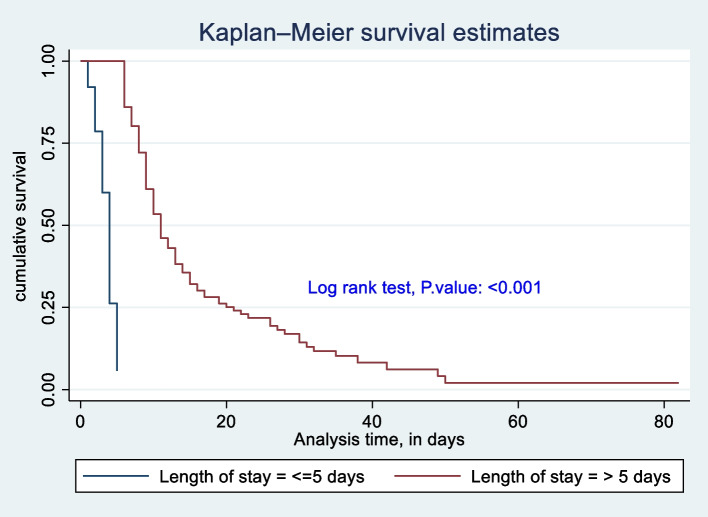
Kaplan-Meier survival estimate of study respondents by length of stay at Dessie Comprehensive Specialized Hospital, North East Ethiopia, 2022

The survival difference among predictor categories was calculated using the log rank test, a *P*-value, and also checked graphically. Predictor categories such as level of consciousness, GCS, time taken for surgical management after admission, type of intent of injury, mechanical ventilation received or not, and length of stay at the hospital had a significant survival difference. Therefore, the log rank test *p*-value of 0.05 and the gap between predictor categories reject the null hypothesis of survival equality (Figs. 4, 5, 6, 7, 8 and 9).

#### Associated predictors of traumatically injured participants

The Cox proportional hazard assumption was not violated as per the graphic test, and the overall global test found an X2 of 19.80 and a *p*-value of 0.4065 (Additional files 2, 3 and 4). Multi-collinearity was not a problem, as VIF and Pearson correlation coefficient resulted in a mean VIF value of 9.66 and a mean Pearson correlation coefficient value of (min: 0.0166 and max: 0.4248).

**Fig. 10:**
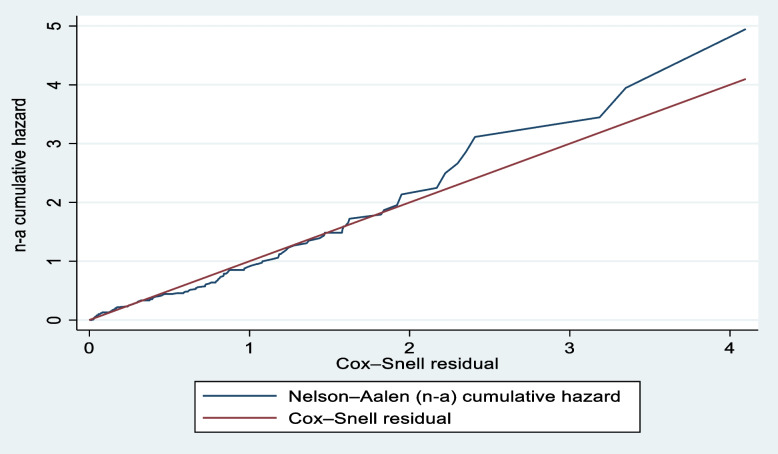
Test of model fitness by using Nelson-Aalen cumulative hazard with Cox-Snell residuals of study respondents at Dessie Comprehensive Specialized Hospital, North East Ethiopia, 2022

The model fitness was estimated through Cox-Snell residuals fitted against Nelson-Aalen cumulative hazard found with a fitted model at straight line and also fitness checked by using Harrell’s C Concordance statistic (found with Harrell’s C value of 69.41%) (Fig. 10).

In multivariable Cox regression analysis, variables such as sex, Glasgow comma scale, time taken for surgical management, intent of injury, mechanisms of injury, and types of TBI were statistically significant for the trauma recovery of participants at 95% CI and a *p*-value of 0.05. On the other hand, age, residency, trauma history, comorbidities, management given before admission, complications present, mode of transport, number of organs injured, site of injury, MV received or not, type of investigations done, and treatment given before ED were not statistically significant.

At any particular time, male respondents were 61.6% (AHR: 0.384, 95%CI: 0.190–0.776, *P*-value: 0.008) less recovered than females. When compared to the GCS group of respondents ≤ 8, research participants in the GCS group of 13- 15 had nearly two and a half times (AHR: 2.563, 95% CI: 1.070–6.139, *P*-value: 0.035) higher rates of trauma recovery.

Similarly, with intent of injury as one independent predictor, those respondents with intentional types of injury had almost two times (AHR: 1.934, 95% CI: 1.03–3.632, *P*-value: 0.040) a faster rate of improvement than those groups with unintentional types of injury. Respondents with RTA injuries had a two-fold (AHR: 2.166, 95% CI: 1.114–4.213, *P*-value: 0.023) increased rate of recovery than participants with non-RTA injuries.

At any given time, research participants with mild TBI had an almost three-fold (AHR: 2.708, 95% CI: 1.095–6.698, *P*-value: 0.031) higher rate of recovery than those with severe TBI. Furthermore, moderate TBI groups recovered twice as well as their counterparts (AHR: 2.253; 95% CI: 1.033–4.911; *P*-value: 0.041) (Table 5).

**Table 5:** Predictors of trauma recovery estimated by using Cox proportional hazard regression of study respondents at Dessie Comprehensive Specialized Hospital, North East Ethiopia, 2022

Predictors	Category	Trauma recovery		CHR (95%CI)	AHR (95%CI)	*P*-value
		Event	Censored			
Sex	Male	200	83	1.13 (0.777–1.644)	0.384 (0.190–0.776)	0.008*
	Female	32	14			
Age	All	32.89 ± 13.91		0.998 (0.989–1.007)	1.002 (0.984–1.019)	0.861
Residency	Urban	107	43	1.018 (0.786–1.318)	0.749 (0.428–1.311)	0.312
	Rural	125	54			
Level of Consciousness	Alert	104	29	2.285 (1.549–3.371)	1.774 (0.638–4.930)	0.272
	Verbally responsive	67	5	2.769 (1.815–4.224)	1.462 (0.545–3.924)	0.450
	Responsive for pain	25	24	1.764 (1.044–2.981)	1.184 (0.350–4.00)	0.786
	Unconsciousness	36	39			
GCS	< = 8	11	41			
	9–12	106	25	4.186 (2.245–7.802)	2.498 (0.994–6.275)	0.051
	13–15	115	31	3.862 (2.077–7.180)	2.563 (1.070–6.139)	0.035*
Previous trauma history	Yes	35	14			
	No	197	83	1.172 (0.814–1.686)	1.656 (0.821–3.337)	0.159
Comorbidities	Yes	11	4			
	No	221	93	1.259 (0.679–2.334)	1.045 (0.319–3.428)	0.942
Management given before admission	Yes	226	93	1.501 (0.667–3.381)	1.792 (0.539–5.95)	0.341
	No	6	3			
Complications present	Yes	62	39			
	No	170	58	1.294 (0.966–1.734)	0.826 (0.484–1.409)	0.483
Time taken for surgical management	< 24 h	169	69	3.082 (1.555–6.109)	3.841 (1.102–13.386)	0.035*
	25–48 h	54	28	1.747 (0.860–3.549)	2.23 (0.609–8.157)	0.226
	> 48 h	9	10			
Intent of injury	Intentional	151	54	1.500 (1.137–1.978)	1.93 4 (1.03–3.632)	0.040*
	Unintentional	81	43			
Mode of transport	Ambulance	152	69	1.128 (0.386–1.128)	1.203 (0.7420–1.951)	0.453
	Non-ambulance	80	28			
Mechanisms of injury	RTA	43	28	0.808 (0.578–1.128)	2.166 (1.114–4.213)	0.023*
	Non-RTA	189	69			
Number of injured organs	Single	189	65	1.229 (0.878–1.721)	0.959 (0.481 1.910)	0.905
	Multiple	43	32			
Site of injury	Head	107	55	0.664 (0.483–0.914)	0.309 (.072–1.328)	0.114
	extremity	26	15	0.515 (0.324–0.819)	0.335 (0.043–2.646)	0.300
	Chest	60	8			
	Abdomen	34	14	0.835 (0.548–1.272)	0.209 (0.032–1.366)	0.102
	Others	5	5	1.011 (0.405–2.525)	0.92 (0.65–2.043)	0.087
Types of TBI	Mild TBI	12	0	3.472 (1.650–7.308)	2.708 (1.095–6.698)	0.031*
	Moderate TBI	70	22	3.546 (2.077–6.056)	2.253 (1.033–4.911)	0.041*
	Sever TBI	27	31			
Received MV	Yes	52	44			
	No	180	53	1.776 (1.297–2.432)	0.77 (0.396–1.500)	0.442
Type of investigations done	Clinical & Laboratory	16	4	1.205 (0.722–2.010)	0.709 (0.267–1.887)	0.491
	Clinical and Imaging	28	20	0.953 (0.640–1.420)	0.479 (0.1851.237)	0.128
	Clinical, Laboratory & imaging	188	73			
Treatment given before ED	Yes	221	95	0.874 (0.476–1.603)	1.04 (0.294–3.686)	0.951
	No	11	2			

^*^Statistically significant at 95%CI & *P*-value < 0.05

## Discussion

A retrospective follow-up study on traumatically injured victims at Dessie Comprehensive Specialized Hospital tried to answer the question of recovery time and its associated predictors for trauma respondents.

As a result, the study found that more than half (70.52%) of respondents had recovered from traumatic injuries, with a confidence interval of 95% (65.34%–75.21%). This finding was lower than studies found in the United States (94.2%) [5], Israel (84%) [18], Italy (80), the Tigray region of Ethiopia (93.8% recovery) [14], and Dilla University Hospital in Ethiopia (94%) [19]. This might be due to the difference in the level of the emergency department and the presence of trauma centers. But, for the highest recovery in other parts of Ethiopia (Tigray region), this is probably due to the fact that in Tigray region, the study was mainly focused on general hospitals, whereas here in this study, it was based on comprehensive specialized hospitals, in which more emergent and complicated cases are referred and further treated. On the other hand, in the Dilla Hospital, Ethiopia, study, it was mainly based on only ED trauma patients, which did not include respondents at the ICU, trauma center, etc., which might lead in to an increase in trauma recovery. Furthermore, while RTA was the most common cause of injury at Dilla University Hospital in Ethiopia, bullet injury was the most common mechanism of injury in this study.

This paper illustrated that the median time of recovery for traumatically injured participants was 5 days (IQR: 3–10 days). This finding was supported by other parts of Ethiopia (the Tigray region), which had 4 days of median recovery [14], and the United States, which had similarly 4 days of recovery. This result was in line with that of a study found in Iran [6], which was 5 days. It was also similarly mentioned in a study in Canada [7] that lasted 6 days. But this finding was lower than that of research conducted at the Urban African Hospital in Kenya [8], which was 24 days. This difference might be due to the fact that the study in Kenya was mainly targeted at a geriatric individuals, which may lead to a slower recovery.

This study also declared that in multivariable Cox proportional regression analysis, variables such as sex, Glasgow comma scale, time taken for surgical management, intent of injury, mechanisms of injury, and types of TBI were statistically significant for the trauma recovery of participants at 95% CI and a *p*-value of 0.05.

At any particular time, male respondents were 61.6% (AHR: 0.384, 95% CI: 0.190–0.776) less recovered than females. However, in a study conducted at Lemlem Karl Hospital in Ethiopia, sex was not identified as a statistically significant predictor. The possible justification might be here in this finding: the major mechanism of trauma was bullet injury, which makes males less improved than females. Participants in the GCS group of 13–15, on the other hand, recovered from trauma nearly two and a half times faster (AHR: 2.563, 95% CI: 1.070–6.139) than those in the GCS group of respondents ≤ 8. This study’s findings are supported by studies conducted at Hadassah University Medical Centre in Jerusalem [20], San Francisco General Hospital in the United States [21], Dilla in Ethiopia [19], and a systematic review of Ethiopian findings [22]. The possible justification might be that the GCS of trauma victims is the hallmark indicator of the level or severity of injury, which has an impact on patient survival.

Similarly, intent of injury was an independent predictor, respondents with intentional types of injuries had an almost two-times (AHR: 1.934, 95%CI: 1.03–3.632) faster rate of improvement than those groups with unintentional type of injury. That is similarly explained with finding at Michigan University, United States [23]. The probable reason for this might be because of unintentional injuries severity of injury as a result of speed of injury. However, mechanism injury with only RTA injury had two times (AHR: 2.166, 95%CI: 1.114–4.213) increased rate of recovery than participants with the rest of injury mechanisms. The possible justification for this might be increased number of bullet injured victims in this study that may be connected with survival.

At any given time, research participants with mild TBI had an almost three-fold (AHR: 2.708, 95%CI: 1.095–6.698) higher rate of recovery than those with severe TBI. Furthermore, moderate TBI groups recovered twice as well as their counterparts (AHR: 2.253, 95% CI: (1.033–4.911). This independent predictor was similarly described in a study done in another part of Ethiopia (Lemlem Karl Hospital) [14]. This might be because the severity of an injury determines the survival of an individual.

### Strengths and limitations of the study

The study’s main strength is that it examines censored variables in the analysis of time to recovery and its predictors for traumatically injured victims. It also calculates the rate of recovery by using the incidence density. In addition, it tries to illustrate the time to recovery of traumatic injuries, for which there are limited studies in the Ethiopian context.

The limitations to be highlighted for this study are its being a single-center study and its nature of being a retrospective follow-up study.

## Conclusion

In conclusion, this study found that more than half of the respondents were recovered. The median time for recovery of traumatically injured research participants was 5 days, with an estimated recovery rate of nine per 100 person-days of observation. Based on the findings of this research, the respective health authorities are recommended to take actions to further increase the recovery time and recovery rate of traumatic injuries. Because, as per this study, even though the average median is 5 days, the highest quartile of time to recovery from injury is up to 10 days. In addition, the proportion of recovery is lower in comparison with most other study findings.

As per the study, the principal reasons for traumatic injuries were bullet injuries, followed by stab injuries and road traffic accidents. It is also advisable to create ways to minimize harm and give priority to those injured with bullet, stab, and RTA injuries since, as per the findings, they are explained as major reasons for trauma and even associated with survival and recovery.

Variables such as gender, Glasgow comma scale, time taken for surgical management, intent of injury, mechanisms of injury, and types of traumatic brain injury were statistically significant with recovery time after trauma in a multivariable Cox proportional regression model.

Similarly, local hospital authorities and health staff, in collaboration with other agencies, should pay special attention to those who are severely injured, focusing on early surgical management (less than 24 h) and reducing the length of stay (less than 5 days) because they demonstrated a survival difference during their stay. Finally, interested researchers are politely invited to further investigate because there is a limited study on time to recovery and its predictors of traumatic injuries in Ethiopia.

### Supplementary Information

**Supplementary Material 1.**

**Supplementary Material 2.**

**Supplementary Material 3.**

**Supplementary Material 4.**

## Data Availability

The dataset used for this research study is available from the corresponding author upon reasonable request.

## Abbreviations

AHR: Adjusted Hazard Ratio
AIDS: Acquired Immune Deficiency Syndrome
CHR: Crude Hazard Ratio
DALY: Disability Adjusted life years
ED: Emergency Department
ED: Emergency Department
GCS: Glasgow Coma Scale
HIV: Human Immunodeficiency Virus
HH: Hazard Ratio
IQR: Inter Quartile Range
KM: Kaplan-Meier
LMIC: Low and Middle Income Countries
LOS: Length of Stay
RTA: Road Traffic Accidents
TBI: Traumatic Brain Injury
WHO: World Health Organization
