# Supplementary material for: Time to recovery and its predictors following traumatic injuries among injured victims in Dessie Comprehensive Specialized Hospital, North East of Ethiopia, 2022: a retrospective follow-up study
Source: BMC Emerg Med. 2024 Mar 18;24:44. doi: 10.1186/s12873-024-00960-9 (PMC10949805; doi:10.1186/s12873-024-00960-9)
Supplement: Supplementary file 1 — Supplementary Material 1. [file 12873_2024_960_MOESM1_ESM.docx]

#### Additional file 1. Sample size determination of the study by using STATA version 16 with stpower log rank through Freedman method

| S/N | Variables | Hazard ratio(HR) | Power | Estimated number of event (E | Estimated initial sample size (n) | Contingency (10%) | Estimated final sample Size (N) | References | Remark |
| --- | --- | --- | --- | --- | --- | --- | --- | --- | --- |
|  | Mechanism of injury | 1.43 | 80 | 252 | 260 | 26 | 286 | (10) |  |
|  | Number of organs involved | 0.42 | 80 | 48 | 58 | 6 | 64 | (13) |  |
|  | Age | 1.38 | 80 | 308 | 320 | 32 | 352 | (10) |  |
|  | Falling down accident & Burn | 1.728 | 80 | 112 | 114 | 12 | 126 | (6) |  |
