# Supplementary material for: Time to recovery and its predictors following traumatic injuries among injured victims in Dessie Comprehensive Specialized Hospital, North East of Ethiopia, 2022: a retrospective follow-up study
Source: BMC Emerg Med. 2024 Mar 18;24:44. doi: 10.1186/s12873-024-00960-9 (PMC10949805; doi:10.1186/s12873-024-00960-9)
Supplement: Supplementary file 3 — Supplementary Material 3. [file 12873_2024_960_MOESM3_ESM.docx]

## Test of cox proportional hazard assumption

### By using Graphs- Log-Log plot of survival

#### Additional file 3: Test of proportional hazard assumption of study respondents by using intent of injury at Dessie Comprehensive Specialized Hospital, North East of Ethiopia, 2022.
