# Supplementary material for: Time to recovery and its predictors following traumatic injuries among injured victims in Dessie Comprehensive Specialized Hospital, North East of Ethiopia, 2022: a retrospective follow-up study
Source: BMC Emerg Med. 2024 Mar 18;24:44. doi: 10.1186/s12873-024-00960-9 (PMC10949805; doi:10.1186/s12873-024-00960-9)
Supplement: Supplementary file 4 — Supplementary Material 4. [file 12873_2024_960_MOESM4_ESM.docx]

## Test of cox proportional hazard assumption

### By using by using Global Schoenfeld test of survival

#### Additional file 4: Test of proportional hazard assumption by using Global Schoenfeld test of study respondents at Dessie Comprehensive Specialized Hospital, North East of Ethiopia, 2022.

| Predictors | Rho | X2 | df | P-value |
| --- | --- | --- | --- | --- |
| Sex | -0.01346 | 0.04 | 1 | 0.8462 |
| Age | 0.01647 | 0.04 | 1 | 0.8474 |
| Residency | 0.01603 | 0.03 | 1 | 0.8531 |
| Level of Consciousness | -0.22697 | 7.78 | 1 | 0.0053 |
| GCS | -0.09764 | 1.18 | 1 | 0.277 |
| Previous trauma history | 0.03602 | 0.2 | 1 | 0.6549 |
| Comorbidities | -0.02106 | 0.06 | 1 | 0.8011 |
| Management given before admission | 0.05263 | 0.32 | 1 | 0.5732 |
| Complications present | -0.07724 | 0.68 | 1 | 0.4097 |
| Time taken for surgical management | -0.14544 | 3.27 | 1 | 0.0704 |
| Intent of injury | -0.0515 | 0.5 | 1 | 0.4809 |
| Mode of transport | -0.08815 | 0.98 | 1 | 0.3222 |
| Mechanisms of injury | -0.05152 | 0.5 | 1 | 0.4794 |
| Number of injured organs | -0.02743 | 0.1 | 1 | 0.7472 |
| Site of injury | 0.11432 | 1.58 | 1 | 0.209 |
| Types of TBI (if head injury) | -0.10322 | 1.5 | 1 | 0.2207 |
| Received MV | -0.19295 | 4.74 | 1 | 0.0296 |
| Type of investigations done | 0.05485 | 0.44 | 1 | 0.5048 |
| Treatment given before ED | 0.09914 | 1.08 | 1 | 0.2981 |
| Global Test |  | 19.8 | 19 | 0.4065 |
